# Construction of an interactive humanistic nursing model in emergency rescue of epidemic outbreaks

**DOI:** 10.12669/pjms.41.1.9705

**Published:** 2025-01

**Authors:** Yaning Feng, Chuntao Ma, Zhongxian Feng, Yajing Bian, Yuqing Leng, Meijin Yuan

**Affiliations:** 1 Yaning Feng Health Management and Physical Examination Center, The First Affiliated Hospital of Hebei North University, Zhangjiakou 075000, Hebei, China; 2 Chuntao Ma Health Management and Physical Examination Center, The First Affiliated Hospital of Hebei North University, Zhangjiakou 075000, Hebei, China; 3 Zhongxian Feng Department of Nursing First School of Clinical Medicine, The First Affiliated Hospital of Hebei North University, Zhangjiakou 075000, Hebei, China; 4 Yajing Bian Department of the Outpatient, The First Affiliated Hospital of Hebei North University, Zhangjiakou 075000, Hebei, China; 5 Yuqing Leng Department of the Outpatient, The First Affiliated Hospital of Hebei North University, Zhangjiakou 075000, Hebei, China; 6 Meijin Yuan Department of Nursing, The First Affiliated Hospital of Hebei North University, Zhangjiakou 075000, Hebei, China

**Keywords:** Epidemic Outbreak, Emergency Rescue, Humanistic Nursing, Compliance, Nursing Quality

## Abstract

**Objective:**

To explore the clinical efficacy of the interactive humanistic nursing model in emergency rescue of epidemic outbreaks.

**Methods:**

This was a retrospective study. A total of 200 patients with novel coronavirus pneumonia (NCP) admitted to The First Affiliated Hospital of Hebei North University between December 2022 and March 2023 were selected and divided into the observation group(n=100) and the control group(n=100) according to different nursing methods used. Patients in the control group received conventional basic nursing intervention, and those in the observation group received humanistic nursing based on basic nursing care. Compared the psychological status, treatment compliance, clinical efficacy, and nursing quality management between the two groups of patients.

**Results:**

The treatment compliance of both groups of patients was significantly improved after intervention, and the degree of improvement in the observation group was higher than that in the control group, with a statistically significant difference (p<0.05). Hospital stay and time to symptom relief and disappearance were shortened in the observation group compared with those in the control group, with statistically significant differences (p<0.05). The scores of nursing efficacy, nursing satisfaction, and nursing safety were increased in the observation group compared with those in the control group, with statistically significant differences (p<0.05).

**Conclusion:**

The interactive humanistic nursing model effectively improves the psychological status of patients and increases treatment compliance, clinical efficacy, and nursing quality, which is worthy of clinical application.

## INTRODUCTION

Epidemic outbreaks refer to public health incidents that occur suddenly and cause or may cause serious damage to public health, which directly endanger people’s life safety and even cause social panic and chaos.^1^,^2^ Epidemic outbreaks are unpredictable and urgent, and a large number of patients may appear in a short period of time with highly complex and varied conditions. The participation of professional medical rescue teams and medical personnel is required.^3^,^4^ Once an outbreak occurs, medical institutes need to quickly implement epidemic prevention measures, bring patients out of danger, and carry out strict nursing management and hospital infection control to control the epidemic within the minimum range in the shortest time and eliminate the threat of social transmission at the fastest speed. With the progress of the times,^5^ the development of transportation, and the convenience of communication, epidemic outbreaks become more frequent.

Outbreaks such as SARS in 2003, the influenza A (H1N1) virus in 2009 and the NCP in 2019 have brought great challenges to medical workers^6^-^8^, especially in the care of patients in epidemic outbreaks. Nursing personnel play an important role in the emergency rescue of epidemic outbreaks, and good nursing quality can reduce the negative impact on patients. They should understand the basic nursing knowledge and the concept of humanistic nursing. Humanistic nursing is a people-oriented nursing concept, which emphasizes that nursing personnel should pay attention to patient’s psychological, social and cultural needs while providing medical services.

The nursing work in emergency rescue involves medical techniques, and more importantly, humanistic care.^9^ The construction of interactive humanistic nursing in response to the outbreak is of great significance to improve rescue effectiveness, soothe the emotions of patients, and protect the mental health of medical staff. In this study, medical records of 100 patients infected with COVID-19 and treated in The First Affiliated Hospital of Hebei North University were retrospectively analyzed to explore the effect of an interactive humanistic nursing model in emergency rescue of epidemic outbreaks.

## METHODS

This was a retrospective study. A total of 200 patients with NCP admitted to The First Affiliated Hospital of Hebei North University between December 2022 and March 2023 were selected and divided into the observation group(n=100) and the control group (n=100) according to different nursing methods used.

### Ethical Approval:

The study was approved by the Institutional Ethics Committee of The First Affiliated Hospital of Hebei North University (No.: K2021071; date: July 16, 2021), and written informed consent was obtained from all participants.

### Inclusion criteria:

- Aged 18 to 70 years old.
- Who met the diagnostic criteria for NCP?
- With normal cognition and mental state, able to communicate and interact with medical professionals normally.
- With complete clinical data.

### Exclusion criteria:

- Expressive language disorders and were unable to communicate normally.
- Mental illness.

### Methods:

Patients in the control group received conventional nursing care, including vital sign monitoring, disease observation and respiratory care, and they were informed of precautions, given diet and exercise instructions, and provided with timely medication or medication instructions according to medical advice, as well as health education. Patients in the observation group received humanistic nursing on the basis of conventional nursing care, as described below:

- A humanistic nursing group was established, and the members of the group received training on the contents, methods and advantages of humanistic nursing to strengthen the awareness of humanistic nursing and improve the understanding of humanistic nursing theory;
- Psychological counseling was provided. Most patients were fear of disease, and some patients experienced serious mental disorders. Therefore, nursing personnel should make efforts to provide patients with psychological counseling, strengthen communication with patients and their families, correct the negative emotions of patients, enhance the education on the knowledge of NCP, and understand the changes of the disease. In addition, they should encourage patients to maintain emotional stability, positive attitude, and optimistic mood, and active
- Construction of humanistic nursing facilities was strengthened in the ward. A clean and tidy environment was provided in the ward to reduce the tension and fear of patients in the unfamiliar environment. Health education was enforced, and consequently, patients understood the necessary knowledge and the treatability of this disease. Problems in the treatment process were timely observed and symptomatic treatment was promptly provided;
- The workflow of humanistic nursing was developed to strictly control the quality of humanistic nursing, focusing on the development of precautions and considerations for admission, treatment and discharge, so that patients could notice the humanistic nursing service throughout the treatment process. Nursing staff can establish a good doctor-patient relationship with patients, win their trust and cooperation. At the same time, a quality control team was set up to regularly evaluate the members of the humanistic nursing team, discover problems timely, and improve the quality of humanistic nursing. During the six-months follow-up of this study, the survival rate was 100%.

### Outcome measures:

- The depression status of patients was assessed using the self-rating depression scale (SDS). This scale included 20 items, with a cutoff score of 53 points, and a higher score indicated more severe depressive symptoms; the anxiety status of patients was assessed using the self-rating anxiety scale (SAS). This scale included 20 items with a cutoff score of 50 points, and a higher score indicated more serious anxiety symptoms; and the patient’s understanding of disease knowledge, preventive measures, precautions and disease observation was assessed using the stress scale. The total score of this scale was 100 points, and a higher score indicated a lower level of stress;
- Treatment compliance: a self-designed patient treatment compliance questionnaire was used to evaluate treatment compliance. The questionnaire included three domains, including compliance with medical advice, dietary plan and daily living activities, with a total of 10 items. A 4-leve
- Clinical efficacy: Hospital stay, and time to symptom relief and disappearance were compared between the two groups;
- Humanistic nursing quality management evaluation: nursing efficacy, nursing satisfaction and nursing safety were evaluated. The total score of the three domains was 100 points, and a higher score suggested a better effect. All the questionnaire was simply handed over to the participants to fill themselves.

### Statistical analysis:

Data analysis was conducted using SPSS22.0 software. The sample size is estimated by 95% confidence interval. Measurement data were presented as (), and two independent samples t tests were used for comparison between groups. Enumeration data were presented as n (%), and χ^2^ test was used for comparison between groups. Differences with a P value of <0.05 were considered statistically significant.

## RESULTS

There were 55 males and 45 females in the observation group, with a mean age of 56.67±9.61 years old (range 40-70 years), and a mean time from onset to admission of 4.20±0.77 days (range 3-5 days), and 53 males and 47 females in the control group, with a mean age of 56.53±9.29 years old (range 40-70 years) and a mean time from onset to admission of 4.19±0.76 days (range 3-5 days). The two groups were comparable with no significant differences in the general data (p>0.05).

There were no significant differences in the SDS, SAS and stress scores between the two groups on admission (p>0.05); and at discharge, the SDS and SAS scores of both groups were significantly decreased compared with those before intervention, and the degree of reduction in the observation group was higher than that in the control group, while the stress score of both groups was significantly increased compared with that before the intervention, and the degree of increase in the observation group was higher than that in the control group, with statistically significant differences (p<0.05) (Table-I).

**Table-I:** Comparison of psychological status before and after treatment between the two groups (*χ̅*±*S*, points).

Groups	SDS score		SAS score		Stress score	

	Admission	Discharge	Admission	Discharge	Admission	Discharge
The observation group(n=100)	57.20±1.39	35.90±2.74	57.43±1.47	39.95±4.09	65.09±5.52	77.77±9.04
The control group(n=100)	57.34±1.43	42.80±2.42	57.52±1.62	42.05±2.28	64.85±6.03	73.63±6.46
*t*	0.728	18.847	0.412	4.488	0.293	3.728
*P*	0.468	0.000	0.681	0.000	0.769	0.000

There was no significant difference in treatment compliance between the two groups before treatment (p>0.05). The treatment compliance of both groups of patients was significantly improved after intervention, and the degree of improvement in the observation group was higher than that in the control group, with a statistically significant difference (p<0.05) (Table-II). Hospital stays and time to symptom relief and disappearance were shortened in the observation group compared with those in the control group, with statistically significant differences (p<0.05) (Table-III). The scores of nursing efficacy, nursing satisfaction, and nursing safety were increased in the observation group compared with those in the control group, with statistically significant differences (p<0.05) (Table-IV).

**Table-II:** Comparison of treatment compliance between the two groups (*χ̅*±*S*, points).

Groups	n	Before treatment	After treatment
The observation group	100	25.63±4.55	35.14±2.91
The control group	100	26.49±3.89	32.25±3.02
*t*		1.437	6.896
*P*		0.152	0.000

**Table-III:** Comparison of clinical efficacy between the two groups (*χ̅*±*S*,d).

Groups	n	Hospital stay	Time to symptom relief	Time to symptom disappearance
The observation group	100	18.02±2.18	7.44±1.10	10.96±0.91
The control group	100	19.51±2.35	8.08±0.63	12.48±0.98
*t*		4.649	5.034	11.374
*P*		0.000	0.000	0.000

**Table-IV:** Comparison of nursing quality management between the two groups (*χ̅*±*S*, points).

Groups	n	Nursing efficacy	Nursing satisfaction	Nursing safety
The observation group	100	84.38±3.23	92.69±3.68	85.25±1.09
The control group	100	81.99±4.03	88.35±4.93	81.64±1.33
*t*		4.624	7.048	21.028
*P*		0.000	0.000	0.000

## DISCUSSION

Patients can feel valued and respected as well as comfort and security. Compared with conventional nursing, the humanistic model of nursing is more comprehensive and detailed, and can provide better nursing care and improved clinical efficacy for patients.^10^ The results of the present study showed that treatment compliance, hospital stay, and time to symptom relief and disappearance were improved in patients receiving humanistic nursing compared with those in patients receiving conventional nursing (p<0.05). Negative emotions such as anxiety and depression may occur in patients with acute infectious diseases due to the uncertainty of clinical outcomes and the need for isolation in treatment, which adversely affect healthy behaviors as well as the physical and mental state of patients, and consequently induce negative emotions such as fear, anxiety, and depression. Once these negative emotions occur, patients may experience low mood, and cannot cooperate with medical professionals during the treatment, which indirectly or directly affects the treatment and outcome of the disease.^11^,^12^

Most epidemic outbreaks are public health incidents that occur suddenly. A large number of patients appear in a short period of time, and their conditions vary, and change and progress rapidly. Clinical rescue for the outbreaks is urgent with high risks, and the results are difficult to predict, with no established diagnosis and treatment plans for reference. If the epidemic is not well controlled in a short period of time, social panic and turmoil may happen, which will cause great social damage.^13^,^14^ The treatment experiences of SARS in 2003 and NCP in 2020 have shown that clinical nursing is an important part of emergency rescue in epidemic outbreaks, and the application of nursing methods directly affects the quality of nursing service.^15^

Nursing care is an important link in response to emergency outbreaks, and the core components of nursing include the observation of disease conditions and the close monitoring of the vital signs of patients to discover the changes in the conditions of patients. Appropriate nursing can improve the treatment compliance of patients, reduce the incidence of complications, and consequently increase the cure rate of disease, reduce mortality, and significantly improve the prognosis of patients.^16^,^17^ Humanistic nursing is a concept of nursing in which patient-centered nursing measures are developed according to the conditions of each individual patient, paying attention to the psychological and physiological needs of patients, to establish a harmonious and warm humanistic nursing environment.^18^

However, humanistic nursing provides specific psychological interventions for different patients, especially positive psychological interventions to reduce the psychological stress responses of patients, and help them to establish positive beliefs, alleviate negative emotions, improve clinical cooperation, and enhance treatment and rehabilitation effects.^19^ In addition, positive energy can be motivated in patients in a positive environment to promote physical and mental relaxation for a better mental and psychological state. The present study showed that the decrease of the SDS and SAS scores and the increase of the stress score were significantly higher in the observation-receiving group with humanistic nursing compared with those in the control group(p<0.05), indicating that the interactive humanistic nursing model can correct the negative emotions in patients.

The humanistic nursing model focuses on the psychology of patients during the implementation process and encourages patients to better understand the disease, which may enable patients to fully know the development, progression, treatment and prognosis of the disease, eliminate their doubts, so that patients can better accept the treatment plan and improve their treatment compliance. Psychological intervention can effectively alleviate the negative emotions of patients, reduce psychological pressure and unhealthy psychology, and establish a correct concept of disease and healthy life behaviors to cooperate with treatment and rehabilitation with a positive and healthy attitude.^20^,^21^ The results of the present study showed that the scores of nursing efficacy, nursing satisfaction and nursing safety of patients in the observation group were increased compared with those in the control group (p<0.05), indicating that the application of interactive humanistic nursing model in emergency rescue can provide patients with multiple psychological and physiological supports, and help to gain the trust of patients and their families. A favorable nurse-patient relationship can be consequently established to improve nursing efficacy, nursing satisfaction and nursing safety.

### Limitations:

It includes small sample size and short follow-up duration, and future studies with a large sample size and a longer follow-up time are needed.

## CONCLUSIONS

The use of an interactive humanistic nursing model in the emergency rescue of epidemic outbreaks effectively soothes the emotions of patients, helps the patient to establish confidence, and improves the psychological quality to cope with epidemics. In addition, it may positively change the mental state and behavior habits and effectively appease the emotions of patients to improve their psychological state and their cooperation with the treatment, which can eventually improve the self-care ability, clinical efficacy and nursing satisfaction of patients. It is an effective strategy to use the interactive humanistic nursing mode in the emergency rescue of epidemic outbreaks.

### Authors` Contribution:

**YF** and **YL:** Carried out the studies, participated in collecting data, and drafted the manuscript, and are responsible and accountable for the accuracy or integrity of the work.

**CM, YB,** **ZF** and **MY:** Literature search**,** Critical Review, Performed the statistical analysis and participated in its design.

All authors have read and approved the final manuscript.
